# Improving the Seebeck Coefficient and Electrical Conductivity of Fe_11_Ti_3_Al_6_ by Substituting Fe with Cr

**DOI:** 10.1007/s11664-024-11723-4

**Published:** 2025-01-10

**Authors:** Sukhwinder Singh, Joseph Alemzadeh, Guillermo Menendez Rodriguez, Matthew Phillips, Daniel Zabek, Matthew Burton, Victoria G. Rocha, Gao Min

**Affiliations:** 1 Magnetics and Materials Research Group, School of Engineering, Cardiff University, Cardiff, CF24 3AA UK; 2 Instituto de Ciencia y Tecnología del Carbono, INCAR-CSIC, Francisco Pintado Fe 26, 33011 Oviedo, Spain; 3 School of Engineering, Swansea University, Swansea, SA1 8EN UK

**Keywords:** Intermetallic, powder metallurgy, thermoelectric, ball milling, spark plasma sintering

## Abstract

In general, any attempt to increase the Seebeck coefficient is usually accompanied by a decrease in the electrical conductivity or vice versa due to the interplay between these two parameters. This work demonstrates that a simultaneous increase in both the Seebeck coefficient and electrical conductivity can be obtained by “doping” in intermetallic alloys. A new alloy composition, Fe_10_Cr_1_Ti_3_Al_6_, was synthesized by substituting Fe with Cr in Fe_11_Ti_3_Al_6_ using mechanical alloying and spark plasma sintering (SPS). The thermoelectric measurements revealed that the Cr substitution led to an increase in the Seebeck coefficient from +27 µV/K in Fe_11_Ti_3_Al_6_ to +39 µV/K in Fe_10_Cr_1_Ti_3_Al_6_, with a corresponding increase in the electrical conductivity from 2.5 × 10^5^ S/m to 4.7 × 10^5^ S/m, resulting in a significant increase in the power factor. The temperature dependence of the thermoelectric properties of this new alloy was also investigated over a temperature range of 50–727°C. The result showed that a maximum power factor of 6.0 × 10^−4^ W/m K^2^ was obtained at 53°C.

## Introduction

A continuous increase in the global energy demand has been seen over the past few decades.^1^ Currently, this demand is largely met by higher consumption of fossil fuels,^2^ leading to increased carbon dioxide emission and global warming.^3^ Reducing fossil fuel consumption is crucial to mitigating global warming.^4^ Thermoelectric (TE) devices can generate electricity directly from waste heat.^5^ They have the potential to make a significant contribution to efficient use of thermal energy and reduction in fossil fuel usage. A key challenge in this field is the development of high-performance thermoelectric materials.^6^ The efficiency of a thermoelectric material can be evaluated based on the dimensionless figure of merit (*ZT*), which is defined as

1 $$ ZT = \frac{{\alpha^{2} T}}{k \rho }, $$

where *α* is the Seebeck coefficient, *ρ* is the electrical resistivity, *k* is the total thermal conductivity, which consists of the electronic (*k*_el_) and lattice (*k*_L_) contribution, and *T* is the absolute temperature. The term $\alpha^{2} /\rho$ is usually referred to as the power factor (PF).

Over the past three decades, thermoelectric materials such as skutterudites,^7^ clathrates,^8^ complex alloys,^9^ Heusler alloys,^10^ metal chalcogenides,^11^ and oxides^12^ have been identified as promising materials, with *ZT* > 1. However, many of these materials contain toxic and costly elements, which limit their usage on a large scale.^13^ To address this problem, scientists have been searching for thermoelectric materials that are abundant, low-cost, and nontoxic.^13^ The efforts in this research direction have resulted in the discovery of promising candidates such as Al-Fe-Si,^14^ Fe-V-Al,^15,16^ and Cu-S.^17^ In particular, due to the presence of the pseudo-gap, the Fe-V-Al alloy^18^ is considered a very promising thermoelectric material, with a power factor of 55 × 10^−4^ W/m K^2^ at room temperature. By implementing the off-stoichiometric concept, Miyazaki et al.^19^ further improved the power factor of the Fe-V-Al alloy to 68 × 10^−4^ W/m K^2^. A similar but less costly Fe_11_Ti_3_Al_6_ alloy was later reported by Garcia-Canadas et al.^20^ which has a room-temperature power factor of 7.0 × 10^−4^ W/m K^2^, with a Seebeck coefficient of +27 µV/K and electrical resistivity of 1 µΩ m. Zou et al.^21^ studied the thermoelectric properties of Fe_2−*x*_Mn_*x*_TiSn (*x* = 0–0.05) and found that Mn substitution improved the power factor and *ZT* compared with Fe_2_TiSn. Fukuta et al.^22^ reported an improvement in the *ZT* of the Fe-V-Al alloy using a grain refinement approach, achieving a high *ZT* value of 0.37. On the other hand, by implementing microstructure engineering, Srinithi et al.^23^ reported an improvement in the power factor of Fe-Al-Si from 2.5 × 10^−4^ W/m K^2^ to 8.8 × 10^−4^ W/m K^2^. Recently, Reumann et al.^24^ investigated the effect of Cr substitution on the Fe site of Fe_2_VAl and obtained a higher Seebeck coefficient of ~70 µV/K in Fe_1.975_Cr_0.025_VAl compared to ~40 µV/K in Fe_2_VAl.

Considering the advantage of the low-cost and nontoxic nature of the Fe_11_Ti_3_Al_6_ alloy, this work was carried out to explore the possibility of further improving its thermoelectric performance. Although Fe_11_Ti_3_Al_6_ exhibits a respectable power factor of 7.0 × 10^−4^ W/m K^2^, its Seebeck coefficient is still too low. Clearly, a strategy for further development of this alloy is to increase its Seebeck coefficient, which may be achieved by adding an additional transition element into the alloy. After initial exploration, we found that the Seebeck coefficient of Fe_11_Ti_3_Al_6_ alloy could be increased by replacing Fe with Cr. In this work, we carried out a focused experimental investigation on the preparation and characterization of the Fe_10_Cr_1_Ti_3_Al_6_ alloy. To the best of our knowledge, this work represents the first attempt to study the material structure and thermoelectric properties of Fe_10_Cr_1_Ti_3_Al_6_ alloy.

## Experimental Methods

Powders of iron, chromium, titanium, and aluminum with 99.9% purity and particle size ranging from 60 µm to 75 µm were purchased from Goodfellow Cambridge Ltd, UK. To prepare Fe_10_Cr_1_Ti_3_Al_6_, 3.42 g of a stoichiometric mixture of these powders was mechanically alloyed in a planetary ball mill (Pulverisette 5/4). The manually blended mixture was loaded under nitrogen atmosphere in a glove box into an 80 mL stainless-steel pot together with stainless-steel balls (10 mm diameter) as the grinding media in a ball-to-powder ratio (BPR) of 35:1. The mechanical alloying process was performed at 300 rpm for 5 h with a 10-min rest after 10 min of milling. The milled powder was then loaded into a 20 mm graphite die set for compaction. The die set was wrapped using graphite felt to minimize heat loss by thermal radiation during consolidation. The milled powders were consolidated into disc-shaped pellets 20 mm in diameter and 1–2 mm thick, using spark plasma sintering (SPS; HP D 10-SD, FCT Systeme GmbH). The SPS cycle was performed in vacuum for 30 min at a heating rate of 100°C/min^25^ and a cooling rate of 50°C/min. Uniaxial pressure was increased linearly up to 48 MPa, maintained during the isothermal stage, and quickly released down to contact force during cooling. The consolidation temperatures were 700°C, 800°C, 900°C, 1000°C, and 1100°C, respectively. One sample was prepared for each consolidation temperature. In addition, Fe_11_Ti_3_Al_6_ samples were prepared to facilitate direct comparison with Fe_10_Cr_1_Ti_3_Al_6_. Although the properties of Fe_11_Ti_3_Al_6_ are available in the literature,^20^ the samples were prepared using a different method. In order to ensure meaningful comparison, both Fe_10_Cr_1_Ti_3_Al_6_ and Fe_11_Ti_3_Al_6_ were synthesized using the same procedures and conditions.

The structure of the sintered samples was analyzed using x-ray diffraction (XRD; Siemens D5000 diffractometer), and the data were processed using X’Pert HighScore Plus software. The density (ρ_*SPS*_) of sintered pellets was measured using the Archimedes method. The chemical composition was determined using scanning electron microscopy/energy-dispersive x-ray spectroscopy (SEM/EDX; Quanta FEG 650). The room-temperature Seebeck coefficient was measured using a hot probe, and the room-temperature electrical resistivity was measured using a multifunctional probe measurement system.^26^ For high-temperature measurements, four samples were prepared using the same procedures as described above, with the consolidation temperature at 1000°C. Two of the samples were cut into bars (3 mm × 3 mm × 10 mm) for the Seebeck and electrical resistivity measurements, and the other two (10 mm in diameter and 3 mm thick) were used for thermal conductivity measurements. The Seebeck coefficient and electrical resistivity were measured in a helium atmosphere using the ULVAC ZEM-3 system over a temperature range of 51–780°C. The thermal conductivity (k) was determined by laser flash measurement (NETZSCH LFA 457 MicroFlash, Germany).

## Results and Discussion

### Crystallization and Phase Transformation

**Fig. 1:**
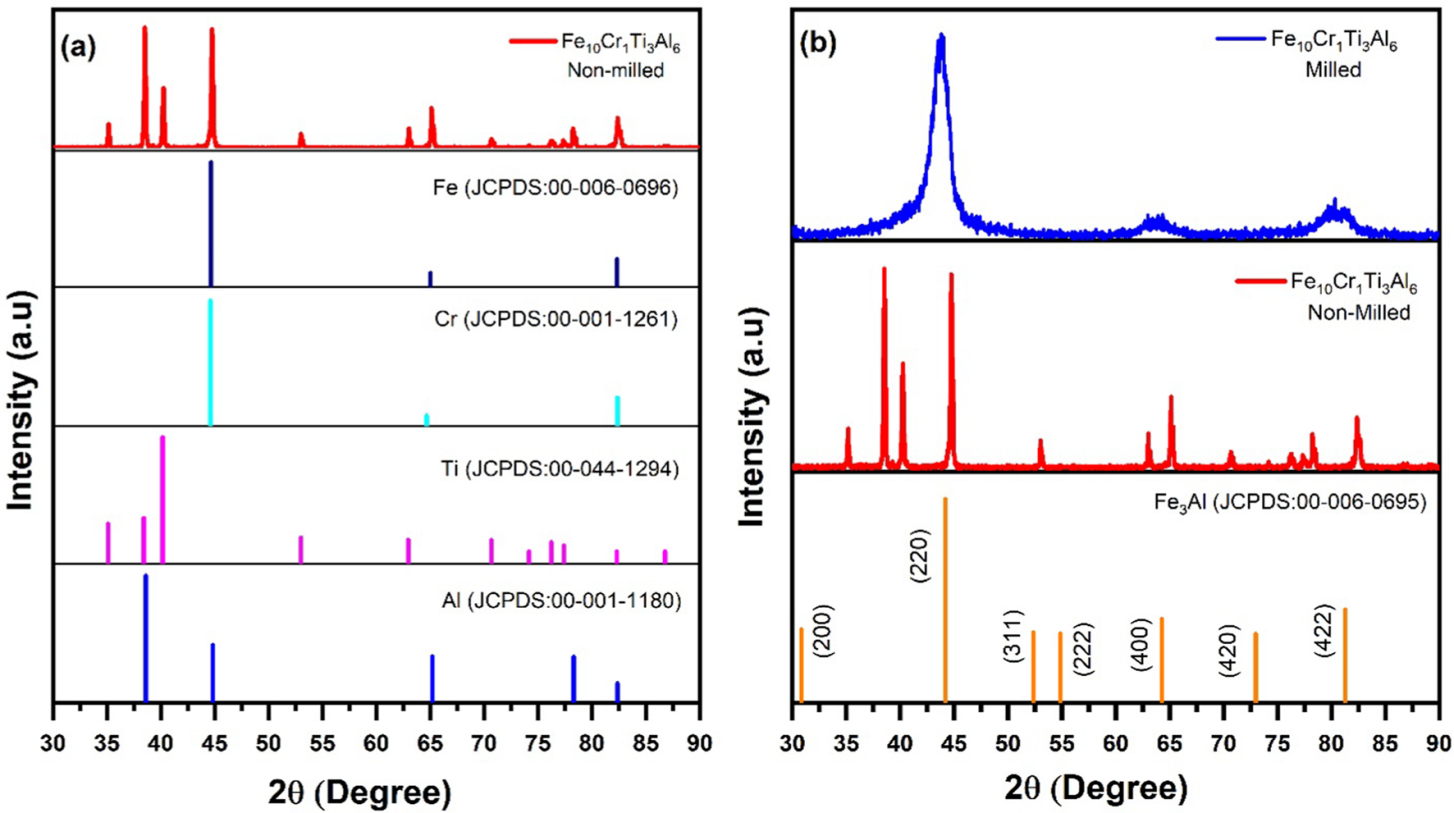
XRD patterns of (a) non-milled powder and (b) milled powder of Fe_10_Cr_1_Ti_3_Al_6_.

To monitor the formation of alloy after ball milling, XRD analysis was used to examine the phase transformation. Figure 1a and b show the XRD results of non-milled and milled powders, respectively. It can be seen that the XRD pattern of non-milled powders shows good agreement with the XRD peaks of elemental Fe (JCPDS/ICDD: 00-006-0696), Cr (JCPDS/ICDD: 00-001-1261), Ti (JCPDS/ICDD: 00-044-1294), and Al (JCPDS/ICDD: 00-001-1180), indicating a pure mixture of elements without alloying. It is interesting to note that three XRD peaks for Fe and Cr are coincidently aligned. After ball milling, the XRD result of the milled powders shows a significantly different pattern, implying a transformation from a pure mixture of elements to an alloy. The position and intensity of the peaks of the milled powder show a XRD pattern similar to Fe_3_Al (JCPDS/ICDD: 00-045-1203), indicating that the milled powders have changed to a phase similar to that of Fe_3_Al. However, it has been reported that it is difficult to obtain the intermetallic phase of (FeTi)_3_Al solely through mechanical alloying.^27,28^ Therefore, it is likely that the ball milling resulted in the formation of Fe(Ti, Cr, Al) solid solution with a body-centered cubic structure as shown by broad peaks. This solid solution might be further transformed into the Fe_3_Al intermetallic phase with an ordered structure after annealing in a temperature range of 350–500°C.^27,28^

**Fig. 2:**
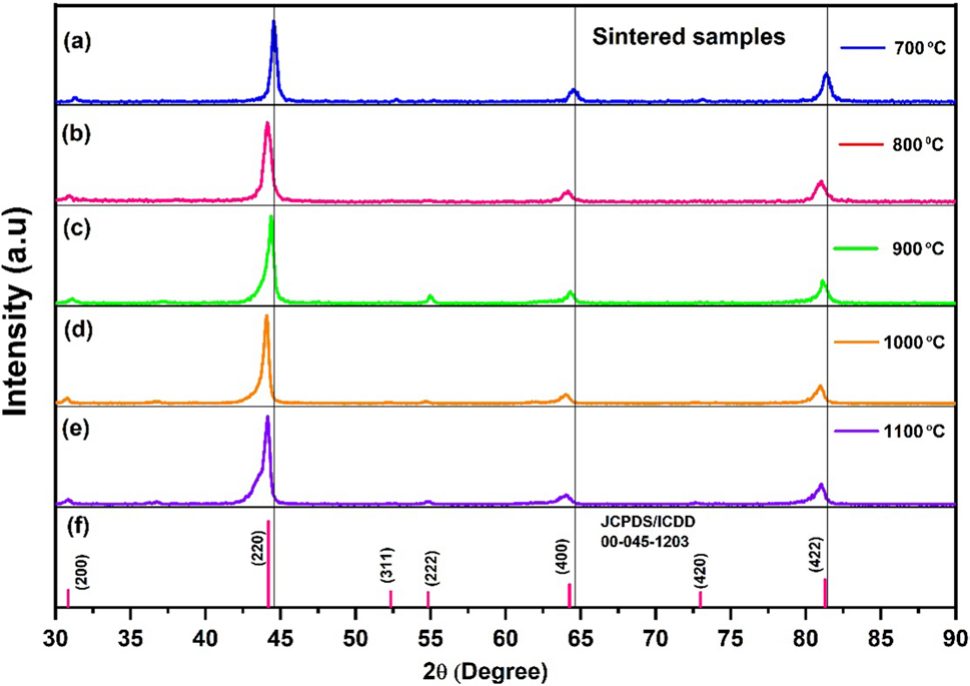
XRD patterns of sintered pellets of Fe_10_Cr_1_Ti_3_Al_6_ at 700°C to 1100°C.

The effect of sintering temperatures on the structural change of the sintered samples was also investigated using XRD analysis. Figure 2 shows the XRD patterns of the sintered pellets at temperatures ranging from 700°C to 1100°C. XRD analysis revealed no formation of new phases after the consolidation of the milled samples at different sintering temperatures. However, the XRD peaks shifted slightly towards the lower angles with increasing sintering temperature, which suggests lattice expansion.^29^ All sintered samples exhibit a crystal structure similar to that of the Fe_3_Al alloy.^30^ In addition, when the sintering temperature was 900°C, a new peak was observed at a diffraction angle of 54.4°, which corresponds to a characteristic peak of the Fe_3_Al intermetallic phase. It is interesting to note that the intensity of this peak then decreased with a further increase in the sintering temperature to 1000°C and 1100°C. This may be associated with the structural transition between the ordered and disordered phases as reported in Fe-Al alloys,^17^ which in turn affects the magnetic properties of materials.^31,32^ However, no correlation could be established between these peaks and the thermoelectric properties. Additionally, no new peaks were identified, suggesting that titanium and chromium were successfully incorporated into the Fe_3_Al structure. However, pinpointing the exact sites of Cr or Ti remains a challenging aspect.

**Fig. 3:**
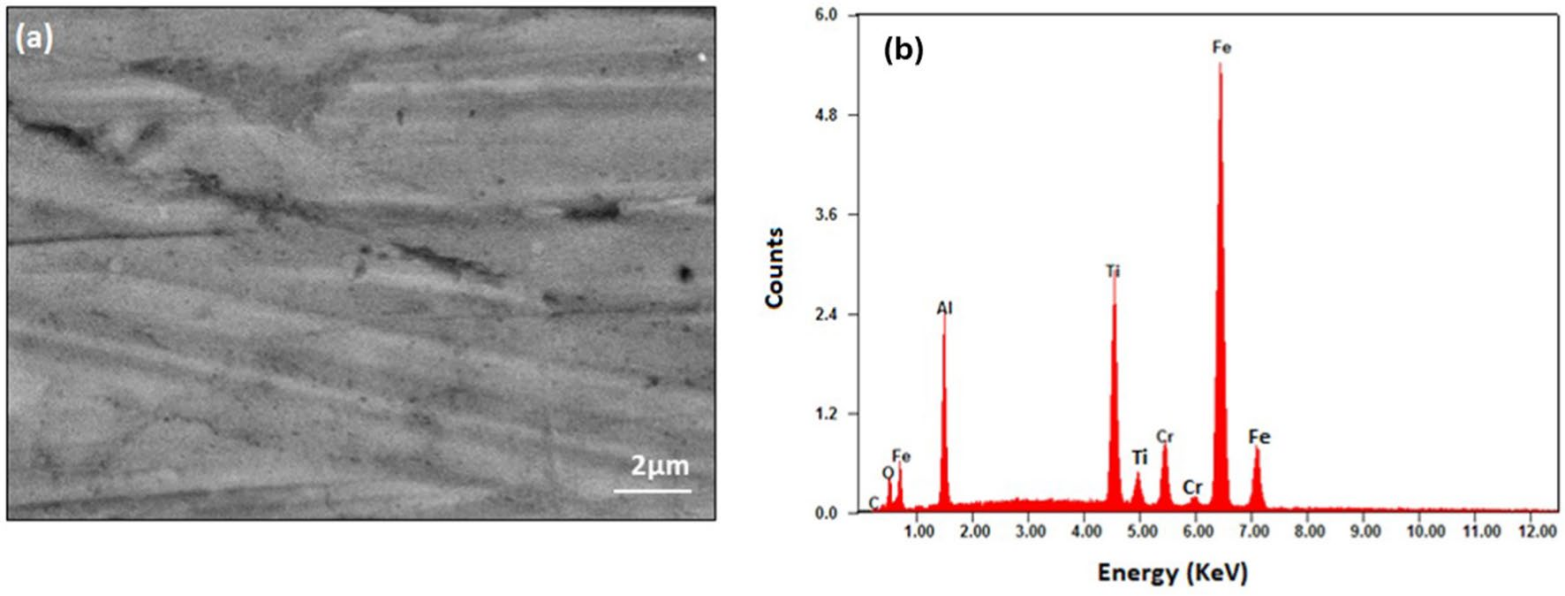
(a) SEM and (b) EDX spectrum of the sample sintered at 1000°C.

Figure 3a and b show the SEM image and EDX spectrum obtained from the polished surface of the sample sintered at 1000°C. EDX analysis was performed to check the chemical composition. The spectrum shows that the sample contains appropriate amounts of Fe, Cr, Ti, and Al. The presence of weak oxygen and carbon peaks indicates minor oxidation and carbon contamination during sample preparation, which is unavoidable and likely to exist at grain boundaries and surfaces. Earlier studies on Fe-Cr alloys^25^ also observed the existence of unexpected elements, potentially linked to the ingress of oxygen from the milling or sintering process.^33^ The chemical composition of the sample from EDX analysis is presented in Table I. It can be seen that all elements added during mixing are retained in the sample after the sintering process. Excluding O and C in Table I, the chemical composition is determined as Fe_9.96_Cr_1.02_Ti_3.00_Al_4.95_, which shows reasonable agreement with the nominal composition of Fe_10_Cr_1_Ti_3_Al_6_. The results clearly show that the appropriate amount of Cr has been added to the alloy, with Fe percentage reduced as expected. The percentage of Al appears to be less than the nominal percentage, indicating that a small amount of Al might be lost during the alloying or sintering process. Nevertheless, the results confirm that the planned “doping” of Cr has been achieved.

**Table I:** Chemical composition of the Fe_10_Cr_1_Ti_3_Al_6_ sample by EDX analysis (the sample was prepared using spark plasma sintering at 1000°C)

Element	Measured weight (%)	Measured atomic (%)	Calculated weight (%) by excluding O and C	Calculated molar amount by excluding O and C
Fe	59.47	44.93	62.79	9.96
Cr	5.67	4.60	5.98	1.02
Ti	15.36	13.53	16.20	3.00
Al	14.23	22.24	15.02	4.95
O	4.36	11.49	–	–
C	0.91	3.21	–	–

### Effect of Sintering Temperature on Density

**Fig. 4:**
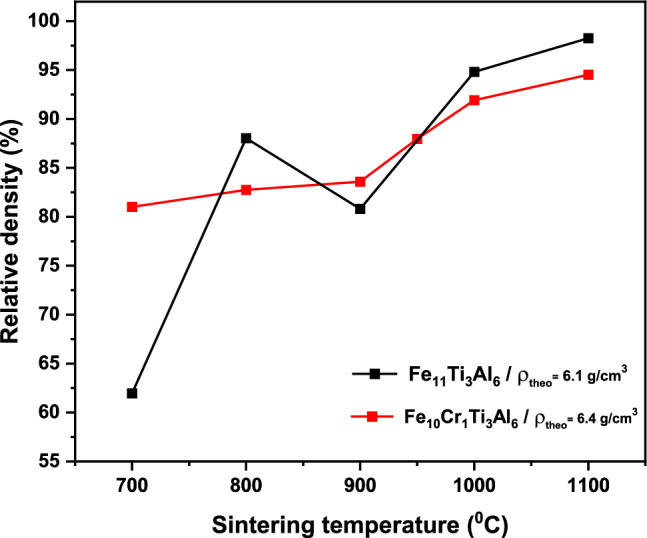
The relative density as a function of sintering temperature for Fe_11_Ti_3_Al_6_ and Fe_10_Cr_1_Ti_3_Al_6_.

SPS was employed to obtain high-density bulk samples. It is anticipated that the density of the samples is strongly depended on the temperature of sintering. Recent studies on Fe-Al intermetallic alloys^34^ have highlighted the effectiveness of the SPS consolidation technique in achieving denser samples at temperatures above 950°C. Figure 4 shows the relative density of Fe_10_Cr_1_Ti_3_Al_6_ sintered samples as a function of sintering temperature. The relative density of the sintered sample was determined using the following equation:

2 $$ \rho_{{{\text{rel}}}} = \frac{{\rho_{SPS} }}{{\rho_{{{\text{theory}}}} }} \times 100, $$

where $\rho_{SPS}$ is the measured density of the sintered samples using the Archimedes method, and $\rho_{{\text{theory }}}$ is the theoretical density of the samples, which was calculated using^35^

3 $$ \rho_{{{\text{theory}}}} = \frac{nM}{{a^{3} N_{A} }}, $$

where *n* is the number of atoms per unit cell of the sample, *M* is the corresponding atomic mass of each element, *a* is the lattice constant, and *N*_A_ is the Avogadro constant (6.023 × 10^−23^ mol^−1^). The calculated density for the sample is 6.4 g/cm^3^. It can be seen from Fig. 4 that the relative density of the samples increases with the increase in sintering temperature. The relative density of the Cr-substituted samples sintered at 900°C is 83%, while the samples sintered at 1000°C and 1100°C possess relative density of 92% and 95%, respectively. This result shows that a higher sintering temperature is preferable for achieving a high-density Fe_10_Cr_1_Ti_3_Al_6_ alloy, consistent with observation in Fe-Al alloys.^34^ An increase in densification with increasing sintering temperature can lead to an improvement in connectivity between grains^36^ and, consequently, the electrical properties of the materials, as shown in the section “Temperature Dependence of Thermoelectric Properties.”

### Influence of Sintering Temperature on the Room-Temperature Thermoelectric Properties

**Fig. 5:**
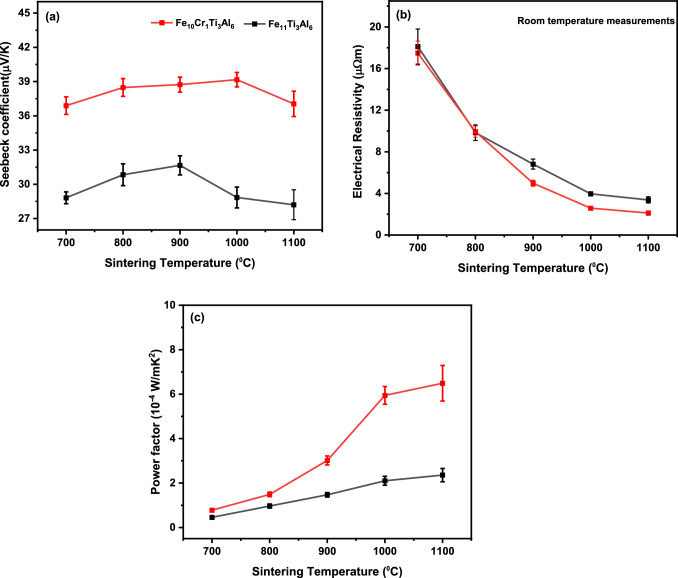
Comparison of room-temperature (a) Seebeck coefficient, (b) electrical resistivity, and (c) power factor for Fe_11_Ti_3_Al_6_ and Fe_10_Cr_1_Ti_3_Al_6_ as a function of sintering temperature.

The room-temperature Seebeck coefficient as a function of the sintering temperature for the Fe_11_Ti_3_Al_6_ and Fe_10_Cr_1_Ti_3_Al_6_ samples is illustrated in Fig. 5a. It can be seen that the Seebeck coefficient of Fe_10_Cr_1_Ti_3_Al_6_ is significantly higher than that of Fe_11_Ti_3_Al_6_ for all sintering temperatures. The highest value of +39 µV/K is obtained from the Fe_10_Cr_1_Ti_3_Al_6_ sample that was sintered at 1000°C. This value is approximately 24% greater than that of the highest Fe_11_Ti_3_Al_6_ sample of this study and about 44% higher than the value of Fe_11_Ti_3_Al_6_ reported in the literature.^20^ This result confirms that the substitution of Fe with Cr can lead to an increase in the Seebeck coefficient. The fact that the Seebeck coefficient is increased due to Cr substitution suggests a shift of the Fermi level towards the density of states where it has a steep slope. This observation can be explained by the Mott formula: $\alpha \left( T \right) \propto \frac{{\partial {\text{N}}\left( {E_{F} } \right)/\partial {\text{E}}}}{{N\left( {E_{F} } \right)}}$, as in the case of Fe-Cr-V-Al^37^ and Fe-V-Al.^16,38^ The Mott formula shows that the Seebeck coefficient is proportional to the derivative of the density of states, $\partial {\text{N}}\left( {E_{F} } \right)/\partial {\text{E}}$. Graphically, it is the slope on the density-of-state plot for a given Fermi level. A steep slope indicates a large value of $\partial {\text{N}}\left( {E_{F} } \right)/\partial {\text{E}}$, corresponding to a large Seebeck coefficient. For a material with its Fermi level located near the trough of the pseudo-gap (such as in Fe_11_Ti_3_Al_6_), shifting the Fermi level away from the trough will increase the slope of the density-of-states plot at the Fermi level and consequently will increase the Seebeck coefficient.

The effect of sintering temperature on the electrical resistivity of the sintered samples is much more significant, as shown in Fig. 5b. The room-temperature electrical resistivity decreases substantially with increasing sintering temperature for both compositions (a factor of 6). This can be attributed to the fact that the electrical resistivity strongly depends on the density of the samples and connectivity at grain boundaries, both of which are improved with increased sintering temperature. The lowest electrical resistivity value of 2.1 µΩ m is obtained in the Fe_10_Cr_1_Ti_3_Al_6_ sample sintered at 1100°C, which is approximately half of that observed in the Fe_11_Ti_3_Al_6_ sintered sample. This reduction in electrical resistivity could be associated with an increase in hole concentration due to Cr substitution, as Cr ([Ar] 3d^5^ 4s^1^) has fewer valence electrons than Fe ([Ar] 3d^6^ 4s^2^), pushing the Fermi energy towards the valence band.^37^ However, the electrical resistivity obtained for the sintered sample remains higher than the electrical resistivity value reported for the Fe_11_Ti_3_Al_6_ composition fabricated using the suspended droplet alloying technique.^20^ This difference is likely associated with the fabrication route.

Figure 5c shows the power factor calculated from the measured Seebeck coefficient and electrical resistivity. It can be seen that the power factor for both Fe_11_Ti_3_Al_6_ and Fe_10_Cr_1_Ti_3_Al_6_ increases with increasing sintering temperature, but it increases more significantly in Fe_10_Cr_1_Ti_3_Al_6_. Such an increase is due to a sharp decrease in the electrical resistivity, while the Seebeck coefficient remains relatively unchanged. The maximum value of 6.4 × 10^−4^ W/m K^2^ was obtained from a Fe_10_Cr_1_Ti_3_Al_6_ sample sintered at 1100°C, which is approximately 2.7 times the highest value from the Fe_11_Ti_3_Al_6_ sintered samples. It is interesting to note that the effect of Cr substitution leads to an increase in both the Seebeck coefficient and electrical conductivity, which rarely happens in conventional semiconductors because a shift in their Fermi level usually leads to an increase in one parameter at the expense of the other. However, in intermetallic alloys, the Seebeck coefficient is strongly dependent on the slope of the density of states ($\partial {\text{N}}/\partial {\text{E}}$) at the Fermi level, rather than on the Fermi level itself. Similar changes in both parameters have also been observed in a Ti-substituted Fe_2_VAl intermetallic alloy.^39^

### Temperature Dependence of Thermoelectric Properties

**Fig. 6:**
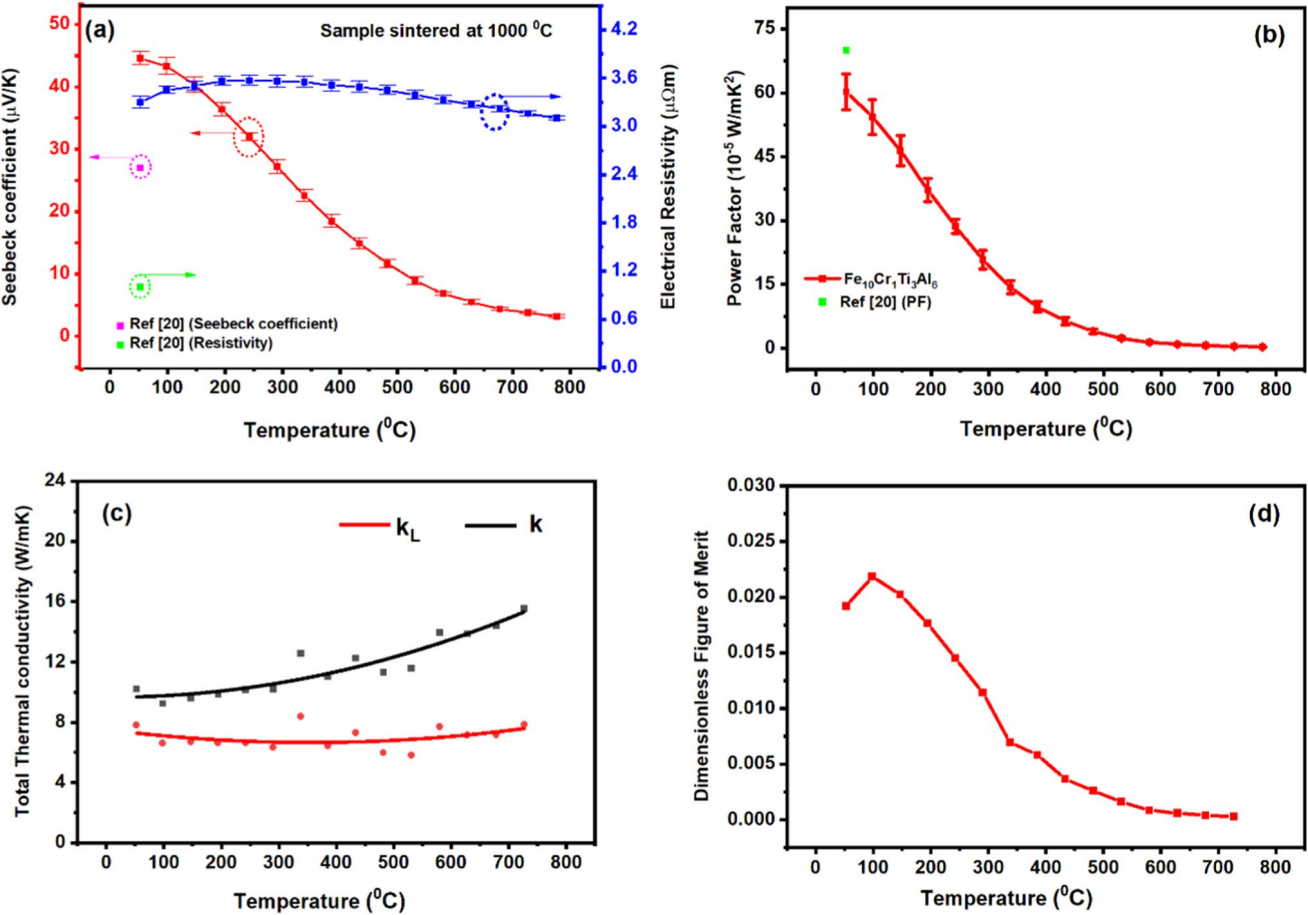
Thermoelectric properties as a function of temperature for the Fe_10_Cr_1_Ti_3_Al_6_ sample sintered at 1000°C. (a) Seebeck coefficient and electrical resistivity, (b) power factor, (c) thermal conductivity, and (d) dimensionless figure of merit.

The sample sintered at 1000°C exhibits the highest Seebeck coefficient among all five samples prepared in this study. It was consequently selected for further investigation of the thermoelectric properties over a temperature range from 50°C to 727°C. Figure 6a shows the Seebeck coefficient and electrical resistivity as a function of temperature for the sample sintered at 1000°C. It can be seen that the Seebeck coefficient decreases quickly with increasing temperature. The largest value of +45 µV/K is obtained at 53°C, which is gradually reduced to 0 µV/K at 800°C. A similar trend was reported in Fe-Mn-Ti-Sn^21^ and Fe_2_TiSn.^40^ The peak value obtained in this work is about 1.7 times that of Fe-Ti-Sn,^21,41^ 1.3 times that of Fe-Mn-Ti-Sn,^21^ and 1.7 times that of Fe_11_Ti_3_Al_6_,^20^ but approximately one third of Al_2_Fe_3_Si_3_^23^ and Fe_2_VAl_1.4_.^42^

The temperature dependence of the electrical resistivity of the sample was measured simultaneously with the Seebeck coefficient along the same direction. The electrical resistivity increased initially with increasing temperature from 3.3 µΩ m at 53°C to 3.6 µΩ m at 242°, and then decreased slightly with a further increase in temperature beyond 242°C. As a result of the sharp decrease in the Seebeck coefficient with temperature, the power factor of the sample also decreased quickly with temperature as shown in Fig. 6b. The fact that the electrical resistivity and the Seebeck coefficient decreased with increasing temperature over the temperature range of 242–776°C indicates that hopping conduction becomes dominant over this temperature range. However, the difference between the temperature (53°C) where the Seebeck coefficient peaks and the temperature (242°C) where the electrical resistivity peaks is much wider than usual. In addition, the fact that the Seebeck coefficient decreases much more quickly than the electrical resistivity is also unusual. Such behavior might be associated with the ferromagnetic properties of the material. Further investigation is needed.

Figure 6c shows the thermal conductivity as a function of temperature, which increases with temperature from 10.2 W/m K at 53°C to 15.5 W/m K at 727°C. The lattice thermal conductivity (k_L_) is also presented, which was determined by subtracting the electronic thermal conductivity (k_e_) from the measured thermal conductivity (k). The electronic thermal conductivity can be calculated using the Wiedemann–Franz law^43^ ($k_{e} = LT/\rho$) and the measured electrical resistivity with *L* = 2.44 × 10^−8^ V^2^K^−2^. It can be seen that the lattice thermal conductivity is approximately 6.0 W/m K and remains more or less constant over the temperature range investigated, while the electronic thermal conductivity increases with temperature due to an increase in the electrical conductivity with temperature. Compared to the established thermoelectric materials, the thermal conductivity of Fe_10_Cr_1_Ti_3_Al_6_ is substantially higher. As a result, its *ZT* value is rather low. Figure 6d shows the *ZT* of Fe_10_Cr_1_Ti_3_Al_6_ as a function of temperature for the sample sintered at 1000°C. The maximum *ZT* value obtained from the study is 0.02 at 98°C. As the temperature increases, the *ZT* decreases quickly due to a reduction in the power factor and an increase in the thermal conductivity. In order to improve the *ZT* of this material, the sharp decrease in the power factor with temperature should be avoided and the lattice thermal conductivity needs to be reduced. It is unusual for the Seebeck coefficient to decrease quickly with temperature while the electrical resistivity remains nearly unchanged. An investigation into this unusual behavior is needed, which can offer valuable insight for improving the power factor of the material. The lattice thermal conductivity may be reduced through grain refinement such as high-pressure torsion processing. The effectiveness of this approach has been demonstrated in Fe-V-Al alloys.^44–46^ In addition, multiple-element doping to form high-entropy alloys can be explored as another promising approach for decreasing the lattice thermal conductivity of the material.^47^

## Conclusion

A new alloy composition, Fe_10_Cr_1_Ti_3_Al_6_, was prepared by replacing Fe with Cr in a Fe_11_Ti_3_Al_6_ alloy using mechanical alloying and spark plasma sintering. Samples with high density of > 90% were obtained at a sintering temperature of ≥ 1000°C under 48 MPa. The EDX and XRD analysis confirmed that the Cr had been incorporated into the alloy as expected, which exhibited a crystal structure similar to Fe_3_Al alloy. The thermoelectric measurements showed that the room-temperature Seebeck coefficient increased from +27 µV/K in Fe_11_Ti_3_Al_6_ to +39 µV/K in Fe_10_Cr_1_Ti_3_Al_6_, and the electrical resistivity decreased from 3.96 µΩ m to 2.1 µΩ m, respectively. Consequently, it resulted in an improvement in the power factor. This is an interesting phenomenon, because a simultaneous increase in both the Seebeck coefficient and electrical conductivity rarely occurs. It is believed that this behavior is associated with the fact that these alloys have a pseudo-gap, in which the Seebeck coefficient is strongly dependent on the slope of the density of state at the Fermi level.

The temperature dependence of the thermoelectric properties was investigated for the sample sintered at 1000°C over a temperature range of 50°C to 780°C. The result showed that the Seebeck coefficient decreased with increasing temperature, whereas the electrical resistivity initially increased with temperature to reach a peak at 242°C, and then decreased with a further increase in temperature. As a result, the maximum power factor of 6.0 × 10^−4^ W/m K^2^ was obtained at 53°C, with a corresponding Seebeck coefficient of +45 µV/K. The thermal conductivity essentially increased with increasing temperature, leading to a peak *ZT* value of 0.02 at 98°C for this alloy.

## Data Availability

Information on the data underpinning the results presented here, including how to access them, can be found in the Cardiff University data catalogue at [10.17035/cardiff.28151768].

## References

[1] A. Zoungrana and M. Çakmakci, From non-renewable energy to renewable by harvesting salinity gradient power by reverse electrodialysis: a review. *Int. J. Energy Res.* 45, 3495 (2021). 10.1002/er.6062.

[2] Ritchie, H. Fossil Fuels. https://ourworldindata.org/energy (2020).

[3] R.B. Jackson, C. Le Quéré, R.M. Andrew, J.G. Canadell, G.P. Peters, J. Roy, and L. Wu, Warning signs for stabilizing global CO_2_ emissions. *Environ. Res. Lett.* (2017). 10.1088/1748-9326/aa9662.

[4] A. Midilli, I. Dincer, and M.A. Rosen, The role and future benefits of green energy. *Int. J. Green Energy* 4, 65 (2007). 10.1080/15435070601015494.

[5] Q. Zhang, Y. Sun, W. Xu, and D. Zhu, Organic thermoelectric materials: emerging green energy materials converting heat to electricity directly and efficiently. *Adv. Mater.* 26, 6829 (2014). 10.1002/adma.201305371. 24687930 10.1002/adma.201305371

[6] C. Gayner and K.K. Kar, Recent advances in thermoelectric materials. *Progr. Mater. Sci.* 83, 330 (2016). 10.1016/j.pmatsci.2016.07.002.

[7] M. Rull-Bravo, A. Moure, J.F. Fernández, and M. Martín-González, Skutterudites as thermoelectric materials: revisited. *RSC Adv.* 5, 41653 (2015). 10.1039/c5ra03942h.

[8] M. Christensen, S. Johnsen, and B.B. Iversen, Thermoelectric clathrates of type i. *Dalton Trans.* 39, 978 (2010). 10.1039/b916400f. 20066182 10.1039/b916400f

[9] G.J. Snyder, and E.S. Toberer, Complex thermoelectric materials. *Nat. Mater.* 7, 105 (2008). 10.1038/nmat2090. 18219332 10.1038/nmat2090

[10] L. Huang, Q. Zhang, B. Yuan, X. Lai, X. Yan, and Z. Ren, Recent progress in half-Heusler thermoelectric materials. *Mater. Res. Bull.* 76, 107 (2016). 10.1016/j.materresbull.2015.11.032.

[11] T.R. Wei et al., Copper chalcogenide thermoelectric materials. *Sci. China Mater.* 62, 8 (2019). 10.1007/s40843-018-9314-5.

[12] G. Ren et al., High performance oxides-based thermoelectric materials. *JOM* 67, 211 (2015). 10.1007/s11837-014-1218-2.

[13] T.R. Wei, C.F. Wu, F. Li, and J.F. Li, Low-cost and environmentally benign selenides as promising thermoelectric materials. *J. Mater.* 4, 304 (2018). 10.1016/j.jmat.2018.07.001.

[14] Y. Takagiwa, Y. Isoda, M. Goto, and Y. Shinohara, Conduction type control and power factor enhancement of the thermoelectric material Al_2_Fe_3_Si_3_. *J. Phys. Chem. Solids* 118, 95 (2018). 10.1016/j.jpcs.2018.03.003.

[15] S. Anand et al., Thermoelectric transport of semiconductor full-Heusler VFe_2_Al. *J. Mater. Chem. C* 8, 10174 (2020). 10.1039/d0tc02659j.

[16] Y. Nishino, *Thermoelectric Energy Conversion 143–156* (Elsevier, 2021).

[17] P. Qiu, X. Shi, and L. Chen, Cu-based thermoelectric materials. *Energy Storage Mater.* 3, 85 (2016). 10.1016/j.ensm.2016.01.009.

[18] H. Okamura, J. Kawahara, T. Nanba, S. Kimura, K. Soda, U. Mizutani, Y. Nishino, M. Kato, I. Shimoyama, H. Miura, K. Fukui, K. Nakagawa, H. Nakagawa, and T. Kinoshita, Pseudogap formation in the intermetallic compounds (Fe_1−x_V_x_)3Al. *Phys. Rev. Lett.* 84(16), 3674 (2000). 10.1103/PhysRevLett.84.3674. 11019174 10.1103/PhysRevLett.84.3674

[19] H. Miyazaki, S. Tanaka, N. Ide, K. Soda, and Y. Nishino, Thermoelectric properties of Heusler-type off-stoichiometric Fe_2_V_1+x_Al_1-x_ alloys. *Mater. Res. Exp.* (2014). 10.1088/2053-1591/1/1/015901.

[20] J. García-Cañadas, N.J. Adkins, S. McCain, B. Hauptstein, A. Brew, D.J. Jarvis, and G. Min, Accelerated discovery of thermoelectric materials: combinatorial facility and high-throughput measurement of thermoelectric power factor. *ACS Combin. Sci.* 18, 314 (2016). 10.1021/acscombsci.5b00178. 10.1021/acscombsci.5b00178 27186664

[21] T. Zou et al., Band structure modification of the thermoelectric Heusler-phase TiFe_2_Sn via Mn substitution. *Phys. Chem. Chem. Phys.* 19, 18273 (2017). 10.1039/c7cp02744c. 28696469 10.1039/c7cp02744c

[22] K. Fukuta, K. Tsuchiya, H. Miyazaki, and Y. Nishino, Improving thermoelectric performance of Fe_2_VAl-based Heusler compounds via high-pressure torsion. *Appl. Phys. A* 128, 1 (2022).

[23] A. Srinithi, H. Sepehri-Amin, Y. Takagiwa, and K. Hono, Effect of microstructure on the electrical conductivity of p-type Fe–Al–Si thermoelectric materials. *J. Alloy. Compd.* 903, 163835 (2022).

[24] Riss, P. D.-I. A., Garmroudi, P. D.-I. F., Parzer, P. D.-I. M. & Reumann, N. Physical properties of Fe_2-x_Cr_x_VAl-type full Heusler compounds. (2021).

[25] A. Kundu, A. Sittiho, I. Charit, B. Jaques, and C. Jiang, Development of Fe-9Cr alloy via high-energy ball milling and spark plasma sintering. *JOM* 71, 2846 (2019). 10.1007/s11837-019-03530-8.

[26] J. García-Cañadas and G. Min, Multifunctional probes for high-throughput measurement of Seebeck coefficient and electrical conductivity at room temperature. *Rev. Sci. Instrum.* (2014). 10.1063/1.4871553. 24784625 10.1063/1.4871553

[27] M. Rafiei, M. Enayati, and F. Karimzadeh, Characterization and formation mechanism of nanocrystalline (Fe, Ti)_3_Al intermetallic compound prepared by mechanical alloying. *J. Alloy. Compd.* 480, 392 (2009).

[28] S.-M. Zhu and K. Iwasaki, Characterization of mechanically alloyed ternary Fe–Ti–Al powders. *Mater. Sci. Eng. A* 270, 170 (1999).

[29] R. Hasan and S.C. Ur, Thermoelectric and transport properties of FeV_1−x_Ti_x_Sb Half-Heusler system synthesized by controlled mechanical alloying process. *Electron. Mater. Lett.* 14, 725 (2018). 10.1007/s13391-018-0088-0.

[30] M.H. Enayati and M. Salehi, Formation mechanism of Fe3Al and FeAl intermetallic compounds during mechanical alloying. *J. Mater. Sci.* 40, 3933 (2005). 10.1007/s10853-005-0718-4.

[31] E.P. Elsukov, E.V. Voronina, A.S. Shuravin, A.V. Zagainov, A.V. Korolev, S.K. Godovikov, E.A. Pechina, and A.E. Elsukova, Structure of the Fe_100-x_Al_x_ alloys (25<x<35 at.%) annealed in a temperature range of 400–800°C and the effect of the ordering type on the magnetic properties. *Phys. Met. Metallograph.* 102, 55 (2006).

[32] A. Mulyawan, T. Terai, and T. Fukuda, Interpretation of Fe-rich part of Fe–Al phase diagram from magnetic properties of A2-, B2-, and DO3-phases. *J. Alloy. Compd.* 834, 155140 (2020).

[33] D. Panda, L. Kumar, and S.N. Alam, Development of Al-Fe_3_Al nanocomposite by powder metallurgy route. *Mater. Today Proc.* 2, 3565 (2015).

[34] O.I. Tolochyn, O.V. Tolochyna, H.A. Bagliuk, Y.I. Yevych, Yu.M. Podrezov, and A.A. Mamonova, Influence of sintering temperature on the structure and properties of powder iron aluminide Fe_3_Al. *Powder Metall. Met. Ceram.* 59, 150 (2020).

[35] L. Huang, R. He, S. Chen, H. Zhang, K. Dahal, H. Zhou, H. Wang, Q. Zhang, and Z. Ren, A new n-type half-Heusler thermoelectric material NbCoSb. *Mater. Res. Bull.* 70, 773 (2015).

[36] X. Chen, A. Weathers, A. Moore, J. Zhou, and L. Shi, Thermoelectric properties of cold-pressed higher manganese silicides for waste heat recovery. *J. Electron. Mater.* 41, 1564 (2012). 10.1007/s11664-012-1987-8.

[37] N. Reumann, A. Riss, F. Garmroudi, M. Parzer, J. Kovacevic, T. Mori, and E. Bauer, Thermoelectric properties and low-temperature transport anomalies in the p-type full-Heusler compounds Fe_2−x_Cr_x_VAl. *Phys. Rev. B* 106(23), 235138 (2022).

[38] B. Hinterleitner, P. Fuchs, J. Rehak, F. Garmroudi, M. Parzer, M. Waas, R. Svagera, S. Steiner, M. Kishimoto, R. Moser, R. Podloucky, and E. Bauer, Stoichiometric and off-stoichiometric full Heusler Fe_2_V_1−x_W_x_Al thermoelectric systems. *Phys. Rev. B* (2020). 10.1103/PhysRevB.102.075117.

[39] H. Matsuura, Doping effects on thermoelectric properties of the pseudogap Fe_2_VAl system. *J. Jpn. Inst. Metals* 66, 767 (2002).

[40] T. Saito, S. Kamishima, and D. Nishio-Hamane, Thermoelectric and magnetic properties of (Fe, Co)_2_TiSn Heusler compounds. *Phys. B Condens. Matter* 639, 413984 (2022).

[41] A. Novitskii, I. Serhiienko, A. Nepapushev, A. Ivanova, T. Sviridova, D. Moskovskikh, A. Voronin, H. Miki, and V. Khovaylo, Mechanochemical synthesis and thermoelectric properties of TiFe_2_Sn Heusler alloy. *Intermetallics* (2021). 10.1016/j.intermet.2021.107195.

[42] M. Parzer, F. Garmroudi, A. Riss, S. Khmelevskyi, T. Mori, and E. Bauer, High solubility of Al and enhanced thermoelectric performance due to resonant states in Fe_2_VAl_x_. *Appl. Phys. Lett.* 120, 071901 (2022).

[43] R.B. Wilson and D.G. Cahill, Experimental validation of the interfacial form of the Wiedemann-Franz law. *Phys. Rev. Lett.* (2012). 10.1103/PhysRevLett.108.255901. 23004623 10.1103/PhysRevLett.108.255901

[44] M. Mikami, Y. Kinemuchi, K. Ozaki, Y. Terazawa, and T. Takeuchi, Thermoelectric properties of tungsten-substituted Heusler Fe_2_VAl alloy. *J. Appl. Phys.* (2012). 10.1063/1.4710990.

[45] S. Masuda, K. Tsuchiya, J. Qiang, H. Miyazaki, and Y. Nishino, Effect of high-pressure torsion on the microstructure and thermoelectric properties of Fe2VAl-based compounds. *J. Appl. Phys.* (2018). 10.1063/1.5034390.

[46] K. Fukuta, K. Tsuchiya, H. Miyazaki, and Y. Nishino, Improving thermoelectric performance of Fe_2_VAl-based Heusler compounds via high-pressure torsion. *Appl. Phys. A* 128, 184 (2022).

[47] S. Ghosh, A. Nozariasbmarz, H. Lee, L. Raman, S. Sharma, R.B. Smriti, D. Mandal, Y. Zhang, S.K. Karan, N. Liu, J.L. Gray, M. Sanghadasa, Y. Xia, S. Priya, W. Li, and B. Poudel, High-entropy-driven half-Heusler alloys boost thermoelectric performance. *Joule* (2024). 10.1016/j.joule.2024.08.008.

